# From cryptic to colorful: Evolutionary decoupling of larval and adult color in butterflies

**DOI:** 10.1002/evl3.149

**Published:** 2019-12-12

**Authors:** Iliana Medina, Regina Vega‐Trejo, Thomas Wallenius, Matthew R. E. Symonds, Devi Stuart‐Fox

**Affiliations:** ^1^ School of BioSciences University of Melbourne Melbourne Victoria 3010 Australia; ^2^ Division of Ecology and Evolution Australian National University Acton Australian Capital Territory 0200 Australia; ^3^ Department of Zoology Stockholm University Stockholm Sweden; ^4^ Centre for Integrative Ecology, School of Life and Environmental Sciences Deakin University Burwood Victoria 3125 Australia

**Keywords:** Butterflies, caterpillars, color, coupled, ontogenetic

## Abstract

Many animals undergo complete metamorphosis, where larval forms change abruptly in adulthood. Color change during ontogeny is common, but there is little understanding of evolutionary patterns in these changes. Here, we use data on larval and adult color for 246 butterfly species (61% of all species in Australia) to test whether the evolution of color is coupled between life stages. We show that adults are more variable in color across species than caterpillars and that male adult color has lower phylogenetic signal. These results suggest that sexual selection is driving color diversity in male adult butterflies at a broad scale. Moreover, color similarities between species at the larval stage do not predict color similarities at the adult stage, indicating that color evolution is decoupled between young and adult forms. Most species transition from cryptic coloration as caterpillars to conspicuous coloration as adults, but even species with conspicuous caterpillars change to different conspicuous colors as adults. The use of high‐contrast coloration is correlated with body size in caterpillars but not adults. Taken together, our results suggest a change in the relative importance of different selective pressures at different life stages, resulting in the evolutionary decoupling of coloration through ontogeny.

**Impact statement**

Most animals in the world undergo metamorphosis, but how do traits evolve before and after this transition? In recent years, there has been interest in understanding what drives the evolution of traits at different life stages. Most of these studies, however, have focused on frogs and/or morphology. An obvious trait that changes through ontogeny in many insects, and that can have large effects on an organism's fitness, is color. Color can be involved in thermoregulation, crypsis, aposematism, and sexual selection. Our study is one of the first to explore and compare how color evolves in butterflies and caterpillars. We use color information on 61% of Australian butterflies and their larvae to investigate whether color evolution is coupled or evolving independently across life stages. We show that (1) adult male coloration is significantly more diverse among species than caterpillar coloration, (2) caterpillars tend to be more cryptic than adults, and (3) color contrast (i.e., how conspicuous a species is) is positively associated with body size in caterpillars but not adults. Our results suggest that crypsis and selection from predators possibly constrain color variation as caterpillars, while sexual selection leads to higher color diversity in male adults. Different selective pressures drive the evolution of color at different points in time, ultimately shaping the evolution of color diversity in this clade.

All animals show phenotypic changes as they age, however, species defined as having complex life cycles (those which undergo metamorphosis) present the most dramatic changes of all. Studying the evolution of a trait across life stages can reveal changes in the relative importance of different selective pressures, and is fundamental to understanding whether selection at early life stages can constrain or facilitate the evolution of adult diversity and phenotypic diversity in general (Roelants et al. 2011; Bonett and Blair 2017; Wollenberg Valero et al. 2017). The “adaptive decoupling hypothesis” suggests that complex life cycles are widespread in animals because such decoupling optimizes performance for stage‐specific tasks in response to changes in selective pressures at different life stages (Moran 1994). For example, the evolution of larval body shape in tadpoles is unrelated to the evolution of shape in adult frogs (Sherratt et al. 2017). Shape is one of the most obvious traits that change during metamorphosis and, hence, one of best studied sources of variation in ontogeny (Wray 1992; Adams and Nistri 2010; Roelants et al. 2011; Katz and Hale 2016; Esquerré et al. 2017). Nevertheless, the many other traits that change through ontogeny have rarely been studied in an evolutionary context.

Color is a striking phenotypic trait with clear adaptive significance, and its role may change dramatically through life with changing selective pressures (Booth 1990; Caro et al. 2016; Cortesi et al. 2016; Salis et al. 2018). For example, in many animals with complete metamorphosis such as butterflies, crabs or frogs, sexual dichromatism develops with the transition to adulthood, when males experience strong sexual selection for conspicuous coloration (Ellers and Boggs 2003; Kronforst et al. 2006; Detto and Backwell 2009; Bell and Zamudio 2012). Similarly, antipredator coloration can change dramatically through life stages as a result of changes in ecology, body size, and behavior. For example, the marine isopod (*Idotea montereyensis)* and the green python (*Morelia viridis*) change color through life to remain cryptic in their environment (Lee 1966; Wilson et al. 2007). The green python changes from red to green to reduce detectability in leafy ground backgrounds early in life or in the canopy in adulthood (Wilson et al. 2007). By contrast, the alder moth (*Acronicta alni*) changes its antipredator strategy and coloration from cryptic brown in early larval stages to black and yellow warning coloration in the last instar when it must actively search for pupation sites (Valkonen et al. 2014). Despite the many well‐documented changes in coloration through ontogeny, we do not know whether coloration in early life stages is coupled with coloration in adulthood, or, specifically, whether color in young predicts adult coloration.

Butterflies (Lepidoptera: Rhopalocera) and their caterpillars are among the most colorful animals in the world. Adults of many species use color in courtship (Kronforst et al. 2006; Oliver et al. 2009), and both life stages use color in thermoregulation and antipredatory strategies (Fields and McNeil 1988; Bowers 1993; Heinrich 1993; Ellers and Boggs 2003). The use of warning signals is very common in both caterpillars and adult butterflies (Nishida 2002; Greeney et al. 2012; Gaitonde et al. 2018), but much less common in pupae (Wiklund and Sillén‐Tullberg 1985; Gaitonde et al. 2018). The propensity to use warning coloration is expected to vary across life stage (Booth 1990; Caro et al. 2016). In early life stages, the size of the signal might be too small or the amount of toxin too low for aposematism to be an effective strategy (Booth 1990; Caro et al. 2016). Younger and smaller stages tend to move more slowly (e.g., are wingless), making crypsis more effective. We would therefore predict that crypsis should be more common than aposematism in caterpillars, which indeed seems to be the case for larval forms in beetles and wasps, which tend to be cryptic (Booth 1990). However, toxicity in caterpillars seems to be common, and in fact in some families of butterflies there is a decrease in toxic compounds (e.g., irioid glycosides) from larval to adult forms (Bowers 1993; Greeney et al. 2012).

Here, we examine the evolution of color in caterpillars and adults in a continent‐wide radiation of Australian butterflies. We quantified the strength of phylogenetic signal in color in both life stages to understand the importance of phylogenetic history in explaining variation across species. Next, we tested which stage exhibits greater color diversity among species by comparing the color space occupied and the variation in color across species within both life stages. We also tested whether color is decoupled across life stages, or whether differences in early stages can predict differences in adulthood. Lastly, we examined the frequency of changes between cryptic and conspicuous color strategies from larval to adult stages. Our results provide a panorama on how color has evolved in each life stage and how different selective pressures, acting at different points in life, have shaped the phenotypic diversity of butterflies and caterpillars across a continent.

## Methods

### DATA COLLECTION

We performed our analysis on 246 species of Australian butterflies for which we found information on both caterpillars and adults, which belong to five families (Papilionidae, Pieridae, Hesperiidae, Nymphalidae, and Lycaenidae) and represent 61% of the total species in Australia. Given that using museum specimens was not an option for caterpillars, we used photographs available on the Internet. We looked for photographs mainly from two websites: https://bobsbutterflies.com.au/ and http://lepidoptera.butterflyhouse.com.au/, but also used other sources employed in other studies on Lepidoptera that have used online photographs (e.g., Kang et al. 2017; Loeffler‐Henry et al. 2019), which are reported in the Supporting Material. These websites are run by amateur lepidopterists and have in most cases high‐quality photos for both butterflies and caterpillars. To ensure that species identification was done properly, we verified the taxonomy using the drawings and pictures in different field guides of Australian butterflies and caterpillars (Orr and Kitching 2010; Braby 2016; James 2017), and we only collected information on latest caterpillar instar available (fifth instar, 246 spp.). For adults, we also used Internet photographs for consistency of approach and to be able to compare the values of both life stages. Using Internet photographs also allowed us to collect information on the underside of the wings, which is difficult to do with delicate museum specimens. For most species, we had a photograph for the caterpillar, the male underside and upperside and the female upperside and underside. We collected color information for males (upperside: 243 spp., underside: 213 spp.) and females (upperside: 153 spp., underside: 119 spp.), but we focus most of our analyses on male upperside color because there was more information available for these.

### EXTRACTION OF COLOR MEASUREMENTS

On each photograph we used the application for Apple Mac—“Digital Colour Meter” to measure standard RGB values, and chose regions of the photograph that represented the different colors present in the organism. We did this manually to ensure that the RGB measurements were done in patches where illumination was appropriate (e.g., no shadows or flash glare) and to ensure that the color measured matched the general perception of color and was not an artifact of the photograph. We sampled between one and four colors per photograph, depending on the number of different colors on the animal. Although the number of colors measured was dependent on the measurer's perception, our analysis is unlikely to be affected by this. First, all data extraction was done by the same person (R.V.‐T.) and second, our analysis is based on color distances, so if a color was accidently ignored (because it was similar to another color) the presence or absence would not affect the results notably, because the distance between that color and the one measured would be low. We assigned each color measured into one of four area categories: (1) primary color (occupies more than 80% of the animal), (2) primary color but shared with another primary color (each occupies between 30% and 50% of the animal), (3) secondary color (10–30%, e.g., thick margin around wings), and (4) secondary color occupying less than 10% of the body of the animal (narrow margins around wing or small spots). We used this procedure rather than quantifying color area because photographs were taken from different angles, and in many cases only half of the caterpillar or only one pair of the wings of the butterfly were visible.

Given that the photographs we used differed in illumination, quality, and angle, our method was a coarse attempt to quantify color in caterpillars and butterflies. Slight differences in RGB values were probably the result of the lack of standardization rather than being meaningful variation useful for our analyses. For this reason, we used the RGB values from the photographs and performed a *K*‐means cluster analysis. This analysis finds natural categories (in our case, color categories) within the data, and assigns each point to one of these categories. We calculated 20 color clusters that were reduced to 17 to minimize redundancy in low reflectance colors (see details in Supporting Material). This method is more objective than assigning manually each color to a pre‐established color category and is more conservative than using the raw values obtained from photographs (since these were not standardized). We acknowledge that our methods ignore the UV component of coloration, which may play an important role in sexual selection in butterflies (Rutowski et al. 2005). Hence, the color distances measured in our analysis likely underestimate true color differences, particularly in adults. Hereafter we refer to the RGB_cluster_ values as the RGB values, which correspond to the cluster coordinates described above. To test the impact of using online photographs, we compared the color distances obtained with online photographs with the distances calculated in a subsample of species for which we had both online photo measurements and standardized photographs for multiple individuals from museum specimens (from Munro et al. 2019). We found a highly significant association between color distances calculated with both types of photographs (detailed in Supporting Material; Figs. S1 and S2).

### MEASURE OF PHYLOGENETIC SIGNAL IN COLORATION AT DIFFERENT LIFE STAGES

We used the multivariate extension of Blomberg's *K* value (*K*_mult_) to quantify the phylogenetic signal in color in each dataset (Adams 2014), using the *physignal* function in the R package “geomorph” (Adams et al. 2017) and a recent phylogenetic analysis of Australian butterflies (Munro et al. (2019), 2500 trees from the posterior probability distribution, details in Supporting Material, *N* = 98 spp.). For each species, we selected the two primary colors (categories 1 and 2) and each color had three associated values (R, G, B). To calculate the multivariate phylogenetic signal, we used the whole array of six dimensions (three per color). Higher values of *K* denote that closely related species are more likely to share similar colors than distantly related species. We arranged the colors such that the first color was always the brightest one (highest R + G + B) and the second one was the darkest one, but when there was only one color then the RGB of the primary color was repeated twice.

### CORRELATION BETWEEN ADULT AND CATERPILLAR COLOR

To test whether differences in color between species as caterpillars can predict differences in color as adults (males and females), we performed a Mantel test in the R package “vegan” (Oksanen et al. 2010). To do this, we first calculated a Euclidean dissimilarity matrix between species within each life stage, for the two primary colors in each photograph (e.g., Euclidean distance with six dimensions), following Sherratt et al. (2017). Colors were arranged so that the brightest (R + G + B) color was always in the first array and the darkest color was in the second array. When there was only one color in a species, then the primary color pattern was used twice to calculate the color distance between species. The Mantel test calculates a Pearson correlation coefficient between adult and caterpillar color distances, and tests the significance by using permutation (10,000 iterations). A significant correlation suggests that species that are similar in color at the caterpillar stage will be similar at the adult stage.

### VISUALIZATION AND COMPARISON OF COLOR SPACE

To be able to visualize the color space occupied by both butterflies and caterpillars, we used a principal component analysis on the RGB coordinates of the two primary colors. These PC axes were not used in any statistical analysis and were only used for visualization in Fig. 2. To compare quantitatively the color space occupied by species in larval and adult forms, we used the RGB values of each color patch for each species and calculated distance in color between different species in this RGB color space. We used three different color distances (A: Euclidean color distance, B: Euclidean area‐weighted distance, C: Earth mover's distance (Weller and Westneat 2019) to ensure that results were repeatable across different distance measures (details in Supporting Material). We computed all the pairwise color distances between species for each of the five datasets (male, female–upper and underside–and caterpillars) and then calculated the mean per dataset and per family within each dataset. This color diversity measure (*D*) represents the average of all the color distances *between species* taking into account all the colors of each species and the area occupied by each color (details in Supporting Material).

We used the *D* value described above to compare the color space occupied by each life stage (e.g., which stage has higher color diversity across species). The difference in *D* value between caterpillars (*D*_caterpillar_) and adults (*D*_adult_) provides information on whether there was ontogenetic change toward more or less diverse colorations across species in adulthood. If *D*_dif_ values (*D*_dif_ = *D*_caterpillar_ – *D*_adult_) are negative, color distances *between species* as adults are larger than color distances between species as caterpillars, implying ontogenetic divergence in color. If *D*_dif_ is positive, then there is more diversity in color in earlier stages, implying convergence in color *across* species in adulthood *sensu* (Esquerré et al. 2017). To assess significance, we ran 1000 permutations (100 for distance B because of computational power) of the data where the *P*‐value was obtained based on the proportion of *D*_dif_ iterated values that were below or above the observed *D*_dif_ (Adams and Nistri 2010). Values significantly lower than the null distribution suggest color divergence in adulthood.

### COMPARISON OF ANTIPREDATOR COLOR STRATEGIES ACROSS LIFE STAGES

To compare how common crypsis and aposematism were across life stages, we coded each species and life stage as belonging to one of three categories: cryptic, contrasting, or ambiguous. This classification was done by calculating the average color distance between a species color pattern and two types of natural backgrounds: a green and a brown one. RGB values from natural green and brown backgrounds were extracted from 10 online photographs. We generated a histogram of the distribution of color distances against natural backgrounds and divided this into three categories with similar breadths (cryptic patterns: 40%, contrasting: 38%, ambiguous: 22%). The combinations of colors in the second group (contrasting) are widely recognized as aposematic signals, and the high contrast of these signals is suggested to have an important role in predator avoidance (Cibulková et al. 2014). We acknowledge that aposematism cannot be confirmed in a species without having information on toxicity and predation, and there are cases where warning colors are not necessarily associated with anti‐predatory defenses (Fields and McNeil 1988; Stamp and Wilkens 1993). These three “color strategy” categories were also correlated to measures of internal contrast in color (e.g., RGB color distance between colors present in the same individual, Fig. S5).

Finally, to evaluate whether the color strategy of the caterpillar was related to the color strategy in the adult, we calculated the percentage of species in each color strategy category. We also tested whether the internal contrasts in adults were correlated with the internal contrast of caterpillars. To do this, we used phylogenetic generalized least squares (PGLS) regression, as implemented in the function *pgls* in the R package “caper’ and used the 2500 phylogenetic trees described previously as the basis for analysis (Orme et al. 2016). The internal contrast of the caterpillar was the predictor and the internal contrast of the adult was the response variable. Only species with phylogenetic information were included (*N* = 98). We extracted the estimate, *t*‐value, and *P*‐value across trees and generated an highest posterior density (HPD) interval for each parameter. To test whether species with warning signals use the same warning signal across life stages, we selected species that had high‐contrast coloration at both life stages, and compared the bright color element of the black + bright color combination. If there was more than one bright color, we used the one that occupied the largest area, if areas were similar we selected the brightest one. We then quantified the percentage of species in which the warning color was the same at both life stages.

For a small subset of 48 species, for which body size data were available from James (2017), we assessed whether body size predicted the degree of internal contrast in the caterpillar and adult (i.e., whether it had contrasting coloration or not), we tested this association statistically using PGLS as described above (but only 29 spp. were included because only these had both body size and phylogenetic information).

## Results

### PHYLOGENETIC SIGNAL AND COLOR SPACE AT DIFFERENT LIFE STAGES

**Figure 1:**
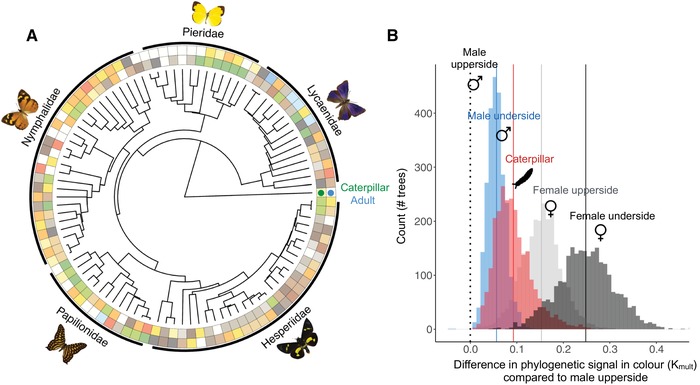
Phylogenetic signal in coloration in Australian butterflies and caterpillars. (A) Maximum clade credibility (MCC) tree showing the primary color (the brightest if there were more than one) in caterpillars (inner circle) and adults (outer circle) for species sampled with phylogenetic information (*N* = 98 spp.). (B) Observed differences between the male upperside phylogenetic signal and male underside (blue), caterpillar (red), and female (light and dark gray) for each of the 2500 different trees. For all trees, the difference is higher than zero, suggesting that male upperside color has the lowest phylogenetic signal regardless of the underlying phylogenetic relationships.

**Figure 2:**
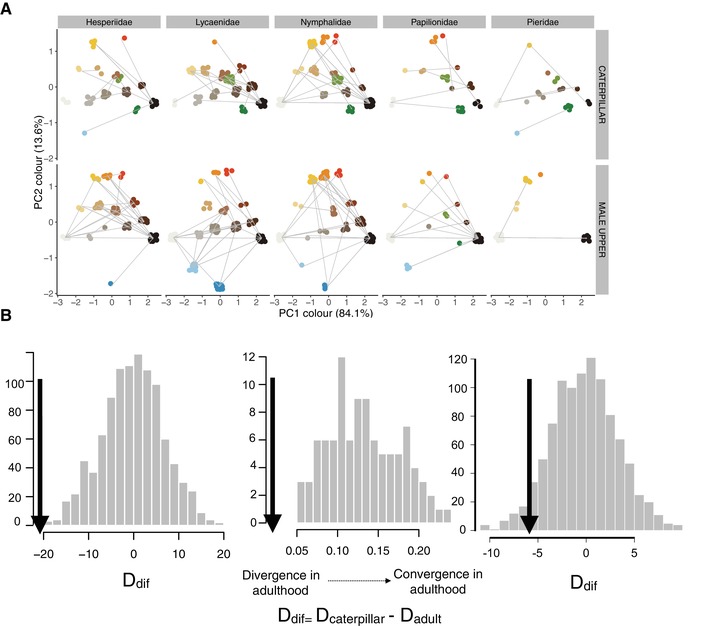
(A) Color space for caterpillars and male upperside coloration across Australian butterflies. Each point represents a primary color (e.g., occupying more than 30% of the wing or caterpillar body). Lines connect colors present as primary colors in the same species. (B) Histograms of randomly expected differences in color diversity (*D*_dif_) between caterpillars and male adults (upperside) using three different color distance metrics (A, B, C described in Supporting Material). Arrows indicate the observed D value (all *P* < 0.05). Values on the left of the random distribution indicate that male adult butterflies are more diverse in coloration than caterpillars across species (ontogenetic divergence). Complete table of results presented in Table S2.

The multivariate measure of phylogenetic signal in color (*K*_mult_) was always higher in caterpillars than in male adults (upperside) across the 2500 possible trees, and lower in male upperside than in female adults and the underside of males (Fig. 1A and B). Distances between species in caterpillar color were not correlated with color distances in adults (Mantel test: *r*_male upperside_ = 0.02, *P* = 0.19; *r*_male underside_ = 0.01, *P* = 0.33; *r*_female upperside_ = 0.04, *P* = 0.93; *r*_female underside_ = 0.03, *P* = 0.23). In general, adults and caterpillars use a wide variety of colors but blue shades are more common in adults than in caterpillars (Fig. 2A). Interspecific diversity in color is higher in adult butterflies than caterpillars, indicating that there is color divergence across species in adulthood (Fig. 2B). These results were consistent across the different methods used to calculate color distances, although when the analysis was done within families results vary slightly across methods (Table S1).

### ANTIPREDATOR COLOR STRATEGIES ACROSS LIFE STAGES

**Figure 3:**
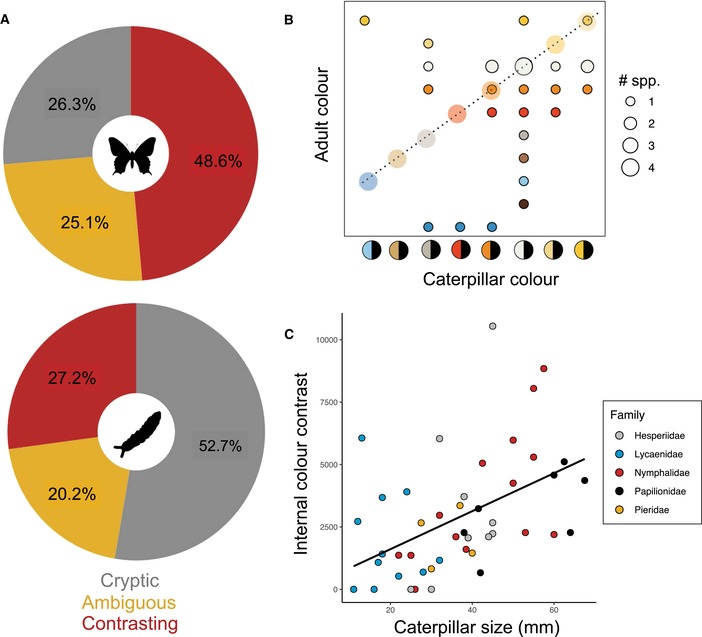
Color strategies in caterpillars and adults. (A) Pie charts showing the percentage of species that employ either of the three anti‐predatory color strategies. (B) Correlation between contrasting colors (the brightest color) used by caterpillars and their adults. If the colors used by the caterpillar and the adult in the same species are the same, then they would follow the diagonal path shown by the dotted line. Only species where both adults and caterpillars have contrasting colorations are shown. (C) Association between caterpillar size and internal contrast in a subset of species (*N* = 48) for which information on caterpillar body size was available.

We found that the most common color strategy in caterpillars is crypsis (Fig. 3A) due to most being either green or brown, while in adults the most common strategy is contrasting coloration due to the common combination of black with a bright color. The most common transition in Australia is from cryptic caterpillars to conspicuous adults (55 spp., 22%) followed by a transition of conspicuous caterpillars to conspicuous adults (40 spp., 16%). In only six of these species (15%), however, is the color used the same in both life stages (Fig. 3B). There was no association between internal contrast in male adults and caterpillars (*N* = 98, PGLS HPD interval across trees for *t* = 0.41 – 0.54, *P* = 0.58 – 0.68). Larger caterpillars have higher internal contrast, and hence are more likely to present warning signals (Fig. 3C, *N* = 29, PGLS HPD interval for *t* = 3.10 – 3.11, *P* = 0.004 – 0.005), but this was not the case for adult dorsal color (*N* = 29, PGLS HPD interval for *t* = 0.99 – 1.00, *P* = 0.33 – 0.34, Fig. S6A). Body size in caterpillars and adults was correlated (*N* = 29, PGLS HPD interval for *t* = 4.64 – 5.06, *P* < 0.005, Fig. S6B).

## Discussion

There is limited information on how traits evolve across different life stages, especially in animals with complex life cycles (Bonett and Blair 2017; Wollenberg Valero et al. 2017). The majority of work in this regard has explored traits associated with morphology (Wray 1992; Katz and Hale 2016; Sherratt et al. 2017; Wollenberg Valero et al. 2017), but there is still a gap in our understanding of how the ontogeny of animal coloration evolves at a broad scale. Here, we measured phenotypic variation in color in both caterpillars and adults in 61% of the butterfly species present in Australia. Overall, we found that color is decoupled across life stages, and color diversity in caterpillars does not predict patterns of color diversity in adults. In other words, two species that have similar‐looking caterpillars will not have similar‐looking adults. We also found that there is larger variation in color in adult butterflies than in caterpillars, and this finding is supported by the larger color distances across species and lower phylogenetic signal in color in male adults compared to the smaller color distances and higher phylogenetic signal in larval stages. In general, butterfly species tend to diverge in color in adulthood. Finally, we found no correspondence between the specific contrasting colors used by caterpillars and adults, and the color strategies used by caterpillars tend to change when they become adults.

Ontogenetic changes in color may occur as a response to the changing selective pressures across life stages. In butterflies, both predation and sexual selection are likely to be the main drivers of wing color in adults, while in larval stages the main selective pressure is probably predation (Heinrich 1993; Oliver et al. 2009). Wing color is an important trait involved in sexual selection in many butterflies, and comparative analyses suggest that the upperside of the wing in males is involved in courtship and female choice, while the color of the underside of the wing is more likely to be driven by predation pressures (Oliver et al. 2009). Interestingly, in our dataset very few caterpillars show blue colors compared to adults. This could be due to constraints on mechanisms of color production in caterpillars, or because blue iridescent colors in adult butterflies are important sexual signals (Rutowski et al. 2005).

Our results support the view that sexual selection is an important driver of butterfly color. First, adult males had higher color diversity across species than caterpillars, and the phylogenetic signal was lower as well, which is expected in traits under selection (Seddon et al. 2008). Second, the dorsal coloration of male adult butterflies presented higher variation in color (and lower phylogenetic signal) compared to the underside of the wing and to female wing coloration. In skippers (Hesperiidae), however, there was consistently similar diversity in butterfly and caterpillar coloration for both males and females, suggesting two possible scenarios. First, sexual selection might not be an important driver of adult color in this family. Alternatively, caterpillars in this family have evolved a higher diversity of colors compared to other families. The first explanation seems more likely, given that both adults and caterpillars in Hesperiidae tend to have inconspicuous, cryptic colors such as brown, which could be an adaptation to the grasslands that these species usually inhabit (Sahoo et al. 2017).

Our results also show that the specific colors that species use are usually different at larval and adult life stages. The majority of caterpillars are cryptic, while most adults tend to be colorful or have contrasting color patterns, that could be used as warning signals. This is also suggested for larvae of beetles and wasps (Booth 1990), and is consistent with the idea that warning colors are not favored in earlier life stages because the toxicity is not high enough, the likelihood of attack is much higher due to the slow movement of larval stages, or the warning signal is not large enough (Booth 1990; Caro et al. 2016). In fact, in our dataset, we found a strong positive relationship between caterpillar size and internal contrast. Internal contrast is higher in species with color combinations usually considered to be warning colors, and this trend supports the idea that body size may predict the evolution of aposematism in caterpillars. It was recently shown that early instar Swallowtail caterpillars tend to be more cryptic than late instars (Gaitonde et al. 2018), and in moths and millipede assassins (Hemiptera: Reduvidae) larger species are more likely to present warning coloration (Kang et al. 2017; Forthman and Weirauch 2018), suggesting this could be a general tendency in aposematic organisms. In contrast to caterpillars, we found no evidence of a link between high color contrast and size in adult butterflies, despite the correlation between caterpillar and adult size. A similar result was found for sea slugs, where there is a negative relationship between size and conspicuousness (Cheney et al. 2014). In butterflies, this could suggest that visual hunting predators may not be the main drivers of high color contrast in adult butterflies, and it is possible that contrast is an important component of sexually selected signals. Alternatively, the relative importance of different selective pressures (predation, sexual selection) may vary according to the species, making it difficult to detect any pattern across the whole dataset.

Only 15% of the species in our dataset were cryptic at both life stages, and an emergent pattern was the frequent transition from green to brown coloration in adulthood. This is probably related to the fact that caterpillars are likely to be camouflaged on stems, while adults are more likely to be camouflaged on the trunks of trees or on leaves (Gaitonde et al. 2018). High‐contrast color patterns are not common in Australian caterpillars, and only 16% of the species in our dataset presented combinations of black and a bright color at both life stages. Of these species, only 15% (6 spp.) presented the same type of color combination in early and adult life stages. If we assume that at least some of these contrasting colorations act as warning signals, our findings suggest that selection does not favor uniformity in color pattern between caterpillars and adults. Naturally, not all contrasting patterns are warning signals, and some warning signals can also be cryptic depending on receiver distance (Barnett et al. 2018).

Ontogenetic changes in warning signals have rarely been explored (but see Gaitonde et al. 2018). There is some evidence that traits under selection are coupled within species before and after metamorphosis in moths and butterflies. For instance, caterpillar color saturation is significantly associated with adult coloration (Lindstedt et al. 2016) and caterpillar diet can significantly affect adult toxicity (Burdfield‐Steel et al. 2018). None of our analyses, however, suggested that color is coupled between life stages across species. Although our sample size is relatively small, because Australia does not have many colorful caterpillars (at least in the butterflies), the very low percentage of species using the same contrasting signals at both life stages suggests that predators are not selecting for uniformity in color between caterpillars and butterflies. In fact, the percentage is so low, that there might even be selection for divergence in warning signals across life stages. This could be due to differences in predators (e.g., invertebrates vs. birds) or because the relative importance of sexual and natural selection favors different color combinations in different life stages (Heinrich 1993). In any case, this finding opens interesting questions regarding the efficacy of warning signals according to body plans, life stage and predator assemblages.

Overall, our results suggest a decoupling of color at the metamorphic boundary, adding to other examples of traits that are decoupled across life stages, such as morphology in frogs (Sherratt et al. 2017; Wollenberg Valero et al. 2017). Our study also hints at how the relative importance of different selective pressures acting at different life stages can generate this decoupling: predation pressure is possibly the strongest driver of caterpillar coloration, while sexual selection is the strongest driver of adult coloration.

Associate Editor: A. Goswami

## Supporting information


**Table S1**. Species added to Munro et al.’s (2019) phylogenetic tree.
**Table S2**. Complete results of *D*_dif_ analyses presented in Fig. 2 in the main text.
**Figure S1**. Comparison between RGB values obtained from online photographs and standardized photographs taken from Munro et al. (2019) for eight species (adult dorsal color) from different families and different color combinations.
**Figure S2**. Color cluster assignment.
**Figure S3**. Validation and comparison of color distances using a sample dataset of eight species (left panel).
**Figure S4**. Two examples of calculation of color distance B between two species.
**Figure S5**. Examples of species in each anti‐predator color category, using color code in B and C.
**Figure S6**. Association between size and internal contrast in adults (left) and caterpillar size and adult size (right).
