# Supplementary material for: From cryptic to colorful: Evolutionary decoupling of larval and adult color in butterflies
Source: Evol Lett. 2019 Dec 12;4(1):34–43. doi: 10.1002/evl3.149 (PMC7006464; doi:10.1002/evl3.149)
Supplement: Supplementary file 1 — Table S1. Species added to Munro et al.’s (2019) phylogenetic tree. Table S2. Complete results of D dif analyses presented in Fig. 2 in the main text. Figure S1. Comparison between RGB values obtained from online photographs and standardized photographs taken from Munro et al. (2019) for eight species (adult dorsal color) from different families and different color combinations. Figure S2. Color cluster assignment. Figure S3. Validation and comparison of color distances using a sample dataset of eight species (left panel). Figure S4. Two examples of calculation of color distance B between two species. Figure S5. Examples of species in each anti‐predator color category, using color code in B and C. Figure S6. Association between size and internal contrast in adults (left) and caterpillar size and adult size (right). [file EVL3-4-34-s001.docx]

**Supplementary material**

**From cryptic to colourful: Evolutionary decoupling of larval and adult colour in butterflies**

**Supplementary Methods**

**K cluster analysis**

Using a clustering approach on the three dimensions (R,G,B) we calculated 20 colour categories using all the colours sampled, and a random starting point set to 1000 to make results repeatable. We visually checked these categories and decided to further merge some categories that had centroids very close to each other in grey and black colours, to be conservative with our measures. This was done because the relationship between RGB and true reflectance values is exponential, meaning that non-linearised RGB values (like the ones we used) tend to inflate differences between dark colours (Stevens *et al.* 2007). We ended up with 17 colour categories which are easily discernible by eye (Figure S2). Each of these categories had an associated coordinate in the RGB space (RGB_cluster_), keeping information on how similar or different are the colour categories.

**Validation of use of photographs**

To test whether colour distances calculated from online photographs were correlated with colour distances from standardised photos we selected eight species for which we had high quality standardised photographs and multiple individuals per species (5 individuals, taken from Munro et al. 2019, details in Munro et al. supplementary material). We measured RGB values for different colour patches (Figure S1) and then calculated colour distances between the eight species using the R package ‘colourdistance’ and the earth mover distance type (Weller & Westneat 2019). We calculated distances for both the online dataset and the standardised photographs. We found that after the cluster analysis the correlation between both distances (online vs. standardised) was high (r^2^= 0.68 to 0.72 – depending on the sample, p always < 0.0001). When we used the raw values of R,G,B the correlation coefficient was between 0.84 and 0.88 (p <0.0001). This indicates that there is a very significant association between the colour distances calculated from online and from standardised photographs. Given that the breadth of colours and species in the real dataset is wider than that tested in this validation (only 8 species), we expect the real correlation to be higher. This analysis supports other studies in which the use of online photographs works well at least when doing broad-scale analyses (e.g. Kang *et al.* 2017; Gaitonde *et al.* 2018; Loeffler-Henry *et al.* 2019), and especially when it is impossible to use museum collections (as is the case for caterpillars).

**Phylogeny**

We used the most recent phylogeny of Australian butterflies (Munro *et al.* 2019), which uses a Bayesian analysis on eight loci and the backbone from Heikkilä *et al.* (2012), and includes 72 of the species present in our dataset, with representatives of all the families in Australia. To this phylogeny we added 26 species that had available phylogenetic information on GeneBank (Table S1), using exactly the same parameters and procedures described in Munro et al (2019). We pruned 2500 trees obtained from the posterior distribution from a Bayesian analysis using BEAST (Drummond & Rambaut 2007) and calculated the highest probability density (HPD) intervals of the phylogenetic signal K_mult_ across the 2500 trees for each life stage using the R package ‘coda’ (Plummer *et al.* 2016), which provide information on phylogenetic uncertainty in the phylogenetic signal and are comparable to 95% confidence intervals but for non-parametric distributions. We used the heatmap function in ‘ggtree’ (Yu *et al.* 2017) and the MCC (maximum credibility tree) to visualise the patterns in a phylogenetic context.

**Calculation of pairwise colour distances and D value**

There are different methods to quantify colour distances between whole colour patterns. We used three different methods that differ slightly in their outcomes. To validate these methods and to understand the differences across them, we generated a sample dataset and we present the outcomes of the comparisons in Figure S3. A description of each of the distance measures used is given below.

*Distance A: Euclidean distances between main colours*

In this first method we calculated Euclidean colour distances only between the main colours of each colour pattern. We selected a maximum of two colours per species (e.g. those colours that occupy more than 30% of area) or one colour, if this occupied more than 80% of the area. We arranged the colours such that the brightest colours were the first in the array and the darkest colours were second. This ensured that the colour distances were being calculated between comparable colours. If there was only one colour pattern then this colour was used for both arrays. After this, an Euclidean distance was calculated between each species, taking into account the six dimensions (r,g and b per colour array). This method is similar to the one used in morphometric analyses (Sherratt *et al.* 2017) implemented in the R package geomorph (Adams *et al.* 2017).

*Distance B: Euclidean distances considering areas*

We calculated a square matrix of all the possible colour Euclidean distances between colour patterns, using the three dimensions (r,g, and b). This matrix was multiplied by a matrix that specified the area occupied by each of the colours compared, such that distances in primary colours were given more importance in the *between species distance* than distances between secondary colours. Because some species had more colours than other species, and we didn’t want larger distances to be correlated with the number of colours measured, we calculated the average colour distance between each pair of species across all their colour patches (see Figure S4 for explanation). We also corrected for the internal contrast within each pattern, so that species with higher internal contrasts did not have higher distances. We then obtained a square matrix containing in each cell the average colour distance (average across colours) across each pair of species in each dataset (246 species for caterpillars and 243 for upper male colouration).

*Distance C: Earth mover’s distance*

This measure is also known as the Wasserstein metric, and it calculates the distance between two distributions as the minimum cost of transforming one colour distribution into another one. It takes into account the area occupied by each colour and the distance in colour space. To calculate this distance we used the recently developed R package colordistance (Weller & Westneat 2019).

*Calculation of D statistic*

To quantify colour variation across all species within caterpillars and within adults (e.g. D value, colour diversity), all the colour distances *between species* were added and this value was divided by the total number of comparisons in the dataset. This ensured that the value obtained for the whole matrix, D, was a total distance measured across all species within each life stage, taking into account all colours in each species and independent of the number of species in the calculation. We called this total colour distance between species D_caterpillar_ or D_adult_. D values were calculated for both datasets (adults and caterpillars) and also within each family in each stage. Higher D values imply higher diversity in colours across species.

**Robustness of results**

We ran pilot analyses using 30 instead of 20 clusters and we found that results were qualitatively identical, so using a different number of clusters or different types of colour distances does not affect our results.

| **Species** | **COI** | **wgl** | **RpS5** | **MDH** | **IDH** | **GAPDH** | **EF1a** | **CAD** | **RpS2** |
| --- | --- | --- | --- | --- | --- | --- | --- | --- | --- |
| *Cephrenes_augiades* | KY019703 | EU364079 |  | KY028019 |  |  | EU364282 | KY045558 | KY028281 |
| *Ocybadistes_walkeri* | KY019827 | EU364075 |  | KY028126 |  | KY027617 | EU364277 | KY045661 | KY028380 |
| *Netrocoryne_repanda* | KY019818 | EU363943 | KY028632 | KY028120 |  | KY027610 | EU364136 | KY045653 | KY028373 |
| *Anisynta_dominula* | KY019664 |  | KY028500 | KY027982 | KY027740 | KY027477 |  | KY045523 | KY028253 |
| *Anthene_emolus* | DQ018946.1 | GQ128912.1 |  |  |  |  | DQ018913.1 | KT286339.1 |  |
| *Antipodia_atralba* | KY019667.1 |  | KY028503.1 | KY027985 | KY027743.1 | KY027480.1 |  | KY045526.1 |  |
| *Carcharodus_alceae* | EU364380.1 | EU363982.1 |  |  |  |  | EU364175.1 |  |  |
| *Cethosia_cydippe* | EU275513.1 | EU275409.1 |  |  |  |  | EU275620.1 |  |  |
| *Cupha_erymanthis* | EU650046.1 | AF014141.1 |  |  |  |  |  |  |  |
| *Doleschallia_bisaltide* | AY788621.1 | AY88496.1 | GQ865423.1 | GQ865199.1 | GQ865086.1 | GQ864955.1 | AY88735.1 |  |  |
| *Elymnias_hypermnestra* | DQ338761.1 | DQ338624.1 |  |  |  |  | DQ338901.1 |  |  |
| *Geitoneura_klugii* | DQ338825.1 | DQ338691.1 | GQ357539.1 |  |  | GQ357409.1 | DQ338973.1 |  |  |
| *Hypocysta_adiante* | EU920738.1 |  | GQ357542.1 |  |  | GQ357412.1 | EU920772.1 |  |  |
| *Neohesperilla_xanthomera* | KF388436.1 | KY019989.1 | KY028630.1 | KY028119.1 | KY027856.1 |  | KY014377.1 | KY045652.1 | KY028372.1 |
| *Oreixenica_lathoniella* | KF399255.1 | GQ357334.1 | GQ357545.1 |  |  | GQ357416.1 | GQ357270.1 |  |  |
| *Pantoporia_consimilis* | MG741085 | MG741920.1 |  | MG741467.1 |  |  | MG741686.1 |  |  |
| *Pelopidas_mathias* | EU364491.1 | EU364083.1 | KY028655.1 | KY028141.1 |  | KY027631.1 | EU364286.1 | KY045675.1 | KY028393.1 |
| *Polyura_schreiber* | GQ256895.1 | GQ256759.1 | GQ257215.1 |  |  |  | GQ257009.1 |  | GQ257096.1 |
| *Praetaxila_satraps* | KT286094.1 |  |  |  |  |  | KT286276.1 | KT286391.1 |  |
| *Sabera_caesina* | EU364488.1 | EU364080.1 |  | KY028179.1 | KY027908.1 | KY027667.1 | EU364283.1 | KY045712.1 | KY028429 |
| *Signeta_flammeata* | KF394140.1 |  | KY028696.1 | KY028186.1 | KY027915.1 | KY027673.1 |  | KY045717.1 | KY028434.1 |
| *Telicota_colon_argeus* | KT582619.1 |  |  | KT582653.1 |  | KY027692.1 |  |  | KY028452.1 |
| *Tirumala_hamata_orientalis* | KP007724.1 | KP007978.1 |  | KP008064.1 |  |  |  |  | KP008107.1 |
| *Vindula_arsinoe* | AY090204.1 | AY090137.1 |  | GQ865278.1 | GQ865157.1 | GQ865048.1 | AY090170.1 | GQ864725.1 | GQ865377.1 |
| *Vagrans_egista* | AY090203 | AY090136 | EU141419 | EU141642 | EU141578 | NO | AY090169 | EU141341 |  |
| *Mydosama_terminus* | DQ338765 | DQ338632 | EU528446 | EU528357 | NO | EU528400 | DQ338905 |  |  |

**‘**

**Table S1.** Species added to Munro et al. 2019 phylogenetic tree. GenBank accession numbers for each gene added.

**Table S2.** Complete results of D_dif_ analyses presented in figure 2 in the main text. Overall and in some of the families there is significant divergence in colour in adulthood, and colours are more diverse in adults compared to caterpillars. P < 0.05 highlighted in bold. Results presented for the three types of colour distances used in the analyses. P Conv represent the P value supporting convergence in adulthood and P Div represents the P value supporting divergence in adulthood.

|  |  |  | **Distance A** |  |  | **Distance B** |  |  | **Distance C** |  |
| --- | --- | --- | --- | --- | --- | --- | --- | --- | --- | --- |
|  |  | D statistic | P Conv | P Div | D statistic | P Conv | P Div | D statistic | P Conv | P Div |
| Male upperside vs. caterpillar | **Combined** | -28.846 | 1.000 | **0.001** | 0.021 | 1.000 | **0.010** | -5.940 | 0.952 | **0.049** |
|  | Papilionidae | -5.156 | 0.588 | 0.413 | 0.506 | 0.660 | 0.341 | 0.083 | 0.011 | 0.99 |
|  | Hesperiidae | 7.075 | 0.314 | 0.687 | 0.077 | 0.389 | 0.612 | 0.055 | 0.075 | 0.926 |
|  | Nymphalidae | 13.299 | 0.141 | 0.860 | -0.030 | 0.750 | 0.251 | 0.053 | 0.012 | 0.989 |
|  | Pieridae | -37.024 | 0.921 | 0.080 | 0.035 | 0.847 | 0.154 | 0.014 | 0.382 | 0.619 |
|  | Lycaenidae | -30.299 | 0.995 | 0.006 | 0.011 | 0.972 | 0.029 | -0.035 | 0.913 | 0.088 |
| Male underside vs. caterpillar | **Combined** | -20.641 | 0.998 | **0.003** | 0.140 | 0.960 | **0.050** | -1.243 | 0.610 | 0.400 |
|  | Papilionidae | -31.190 | 0.919 | 0.082 | 0.603 | 0.743 | 0.258 | 0.024 | 0.205 | 0.796 |
|  | Hesperiidae | 0.981 | 0.441 | 0.560 | 0.037 | 0.354 | 0.647 | 0.023 | 0.215 | 0.786 |
|  | Nymphalidae | 12.233 | 0.153 | 0.848 | -0.120 | 0.848 | 0.153 | 0.040 | 0.019 | 0.982 |
|  | Pieridae | -47.656 | 0.992 | 0.009 | 0.507 | 0.407 | 0.594 | -0.028 | 0.780 | 0.221 |
|  | Lycaenidae | -32.639 | 0.997 | 0.004 | 0.154 | 0.991 | 0.010 | -0.028 | 0.798 | 0.203 |
| Female upperside vs. caterpillar | **Combined** | -12.746 | 0.912 | 0.089 | 0.013 | 1.000 | **0.010** | 0.536 | 0.470 | 0.540 |
|  | Papilionidae | 46.451 | 0.013 | 0.988 | 0.462 | 0.458 | 0.543 | 0.142 | 0.002 | 0.999 |
|  | Hesperiidae | 19.854 | 0.204 | 0.797 | 0.207 | 0.146 | 0.855 | 0.066 | 0.087 | 0.914 |
|  | Nymphalidae | 21.704 | 0.052 | 0.949 | -0.221 | 0.787 | 0.214 | 0.056 | 0.014 | 0.987 |
|  | Pieridae | -36.883 | 0.890 | 0.111 | -0.042 | 0.884 | 0.117 | -0.002 | 0.472 | 0.529 |
|  | Lycaenidae | -21.243 | 0.966 | 0.035 | 0.081 | 0.982 | 0.019 | -0.015 | 0.651 | 0.350 |
| Female underside vs. caterpillar | **Combined** | -7.819 | 0.657 | 0.344 | 0.180 | 0.287 | 0.714 | 4.651 | **0.037** | 0.964 |
|  | Papilionidae | 4.188 | 0.353 | 0.648 | 0.472 | 0.455 | 0.546 | 0.072 | 0.024 | 0.977 |
|  | Hesperiidae | 34.941 | 0.130 | 0.872 | -0.060 | 0.208 | 0.793 | 0.106 | 0.042 | 0.959 |
|  | Nymphalidae | 24.248 | 0.037 | 0.964 | -0.268 | 0.777 | 0.224 | 0.065 | 0.005 | 0.996 |
|  | Pieridae | -50.291 | 0.990 | 0.011 | 0.544 | 0.232 | 0.769 | -0.041 | 0.764 | 0.237 |
|  | Lycaenidae | -21.839 | 0.854 | 0.147 | 0.282 | 0.642 | 0.359 | -0.008 | 0.378 | 0.623 |

**Supplementary Figures**

**
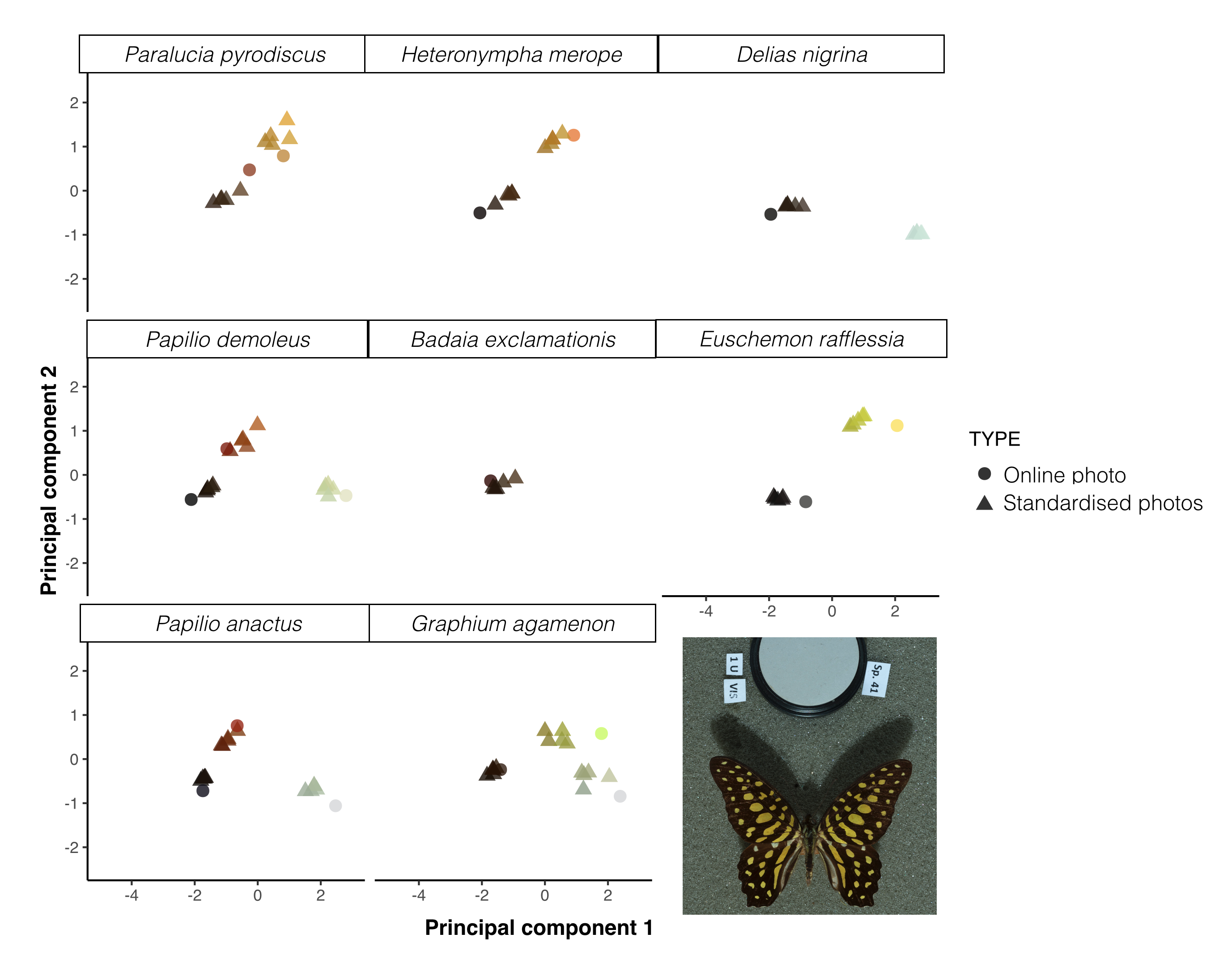
**

**Figure S1.** Comparison between RGB values obtained from online photographs and standardised photographs taken from Munro et al. 2019 for eight species (adult dorsal colour) from different families and different colour combinations. Details on specifications for photographs in supplementary material of Munro et al. 2019. Example of standardised photograph of *Graphium agammenon.*


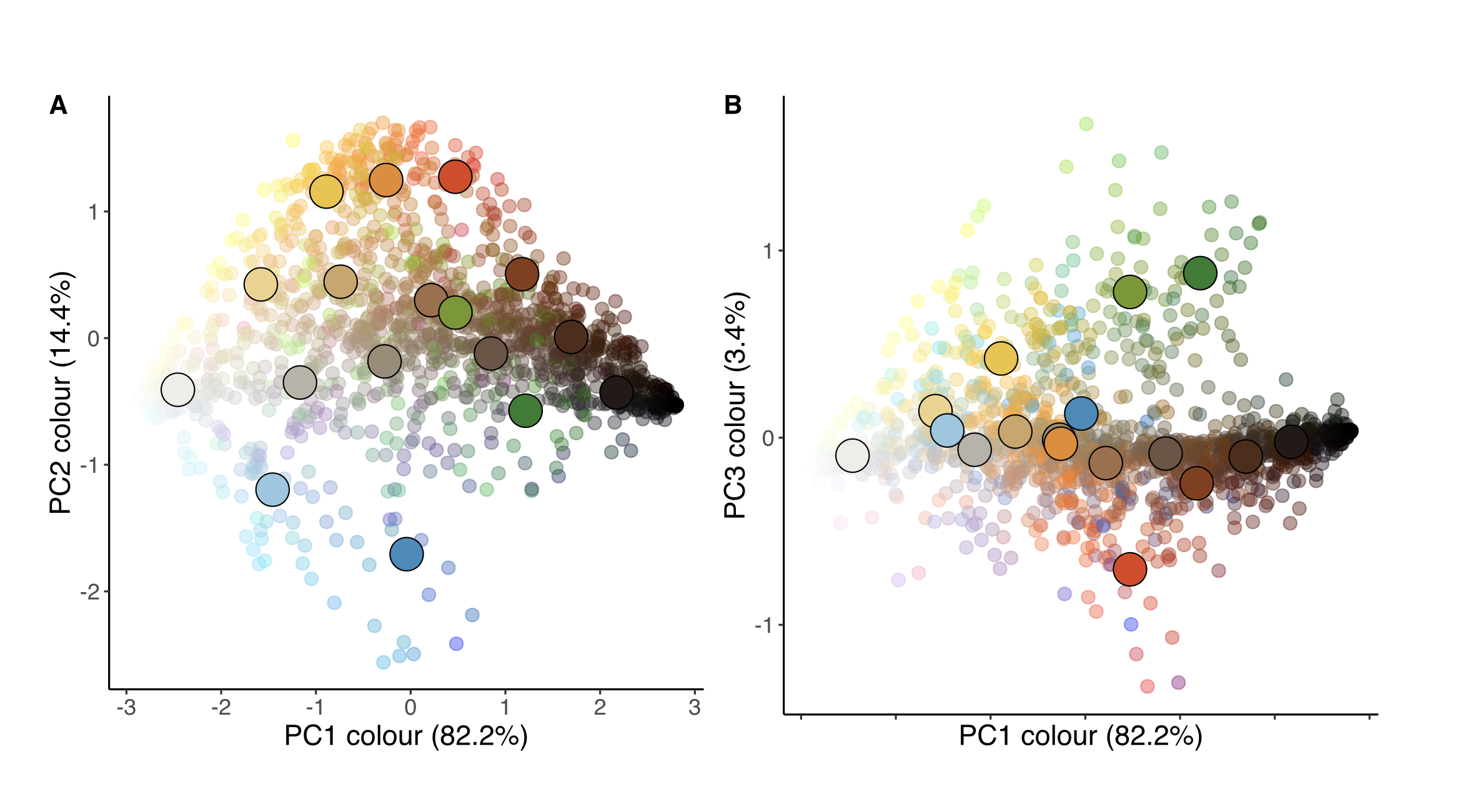


**Figure S2.** Colour cluster assignment. We used a K-means algorithm to assign each RGB colour measured to one of 17 natural colour categories in our dataset (large points). Background transparent points represent actual colours measured.


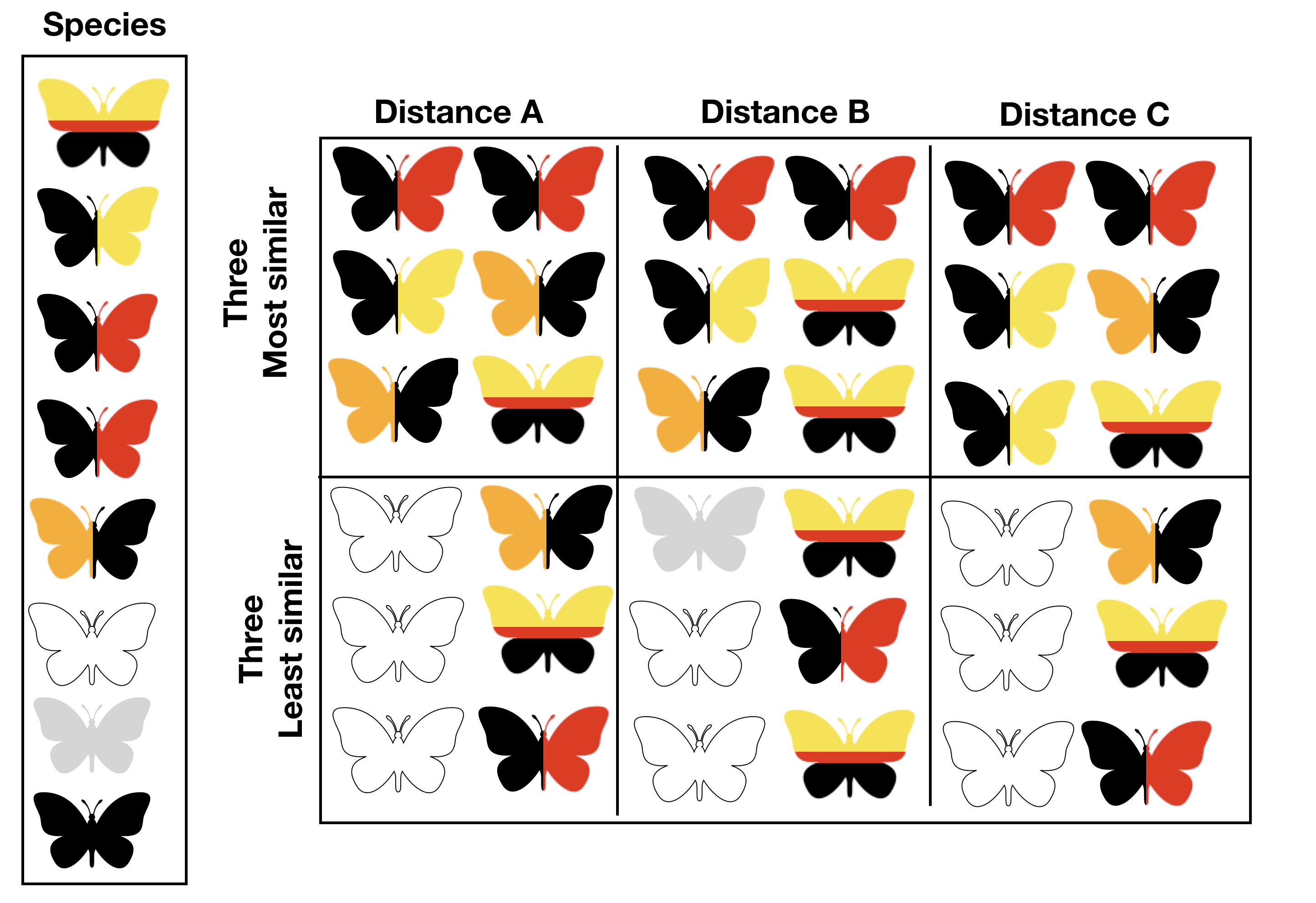


**Figure S3**. Validation and comparison of colour distances using a sample dataset of eight species (left panel). On the right panel we show on the top the three most similar pairs of species (in order) according to each distance measure. Figures on the bottom show the most different species. There are similarities in the three types of distances but there are also slight differences. For example, distance B considers that extra colours increase differences between species.


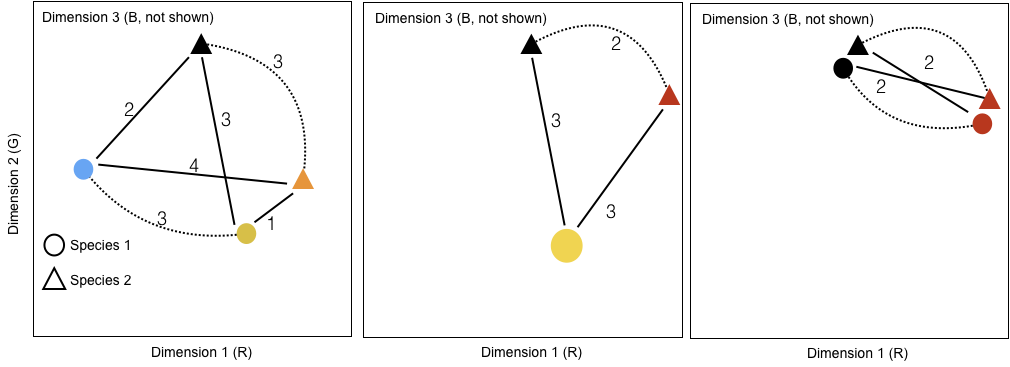


**Figure S4.** Two examples of calculation of colour distance B between two species. Left figure shows two species with two colours each, and right one shows two species, one with only one colour. The Euclidean distances using three dimensions (R,G and B) were calculated between the colours of each species (numbers on top of lines). The size of the circle and triangle represents the area occupied by that colour (either 8 (80%) or 5 (50%)). Each distance between colours in different species was multiplied by the sum of areas in both colours (e.g. for diagram in the middle: 3*(8+5) + 3*(8+5)) and divided by the number of distances calculated (e.g. 78/2 = 39). In this way, distances in main colours are weighted higher than distances in secondary colours. After this, we corrected (e.g. subtracted) for the average internal contrast of each species (39 – (2*(5+5)+0)/2=29). This ensured that the total distance between the species in the third panel is zero.


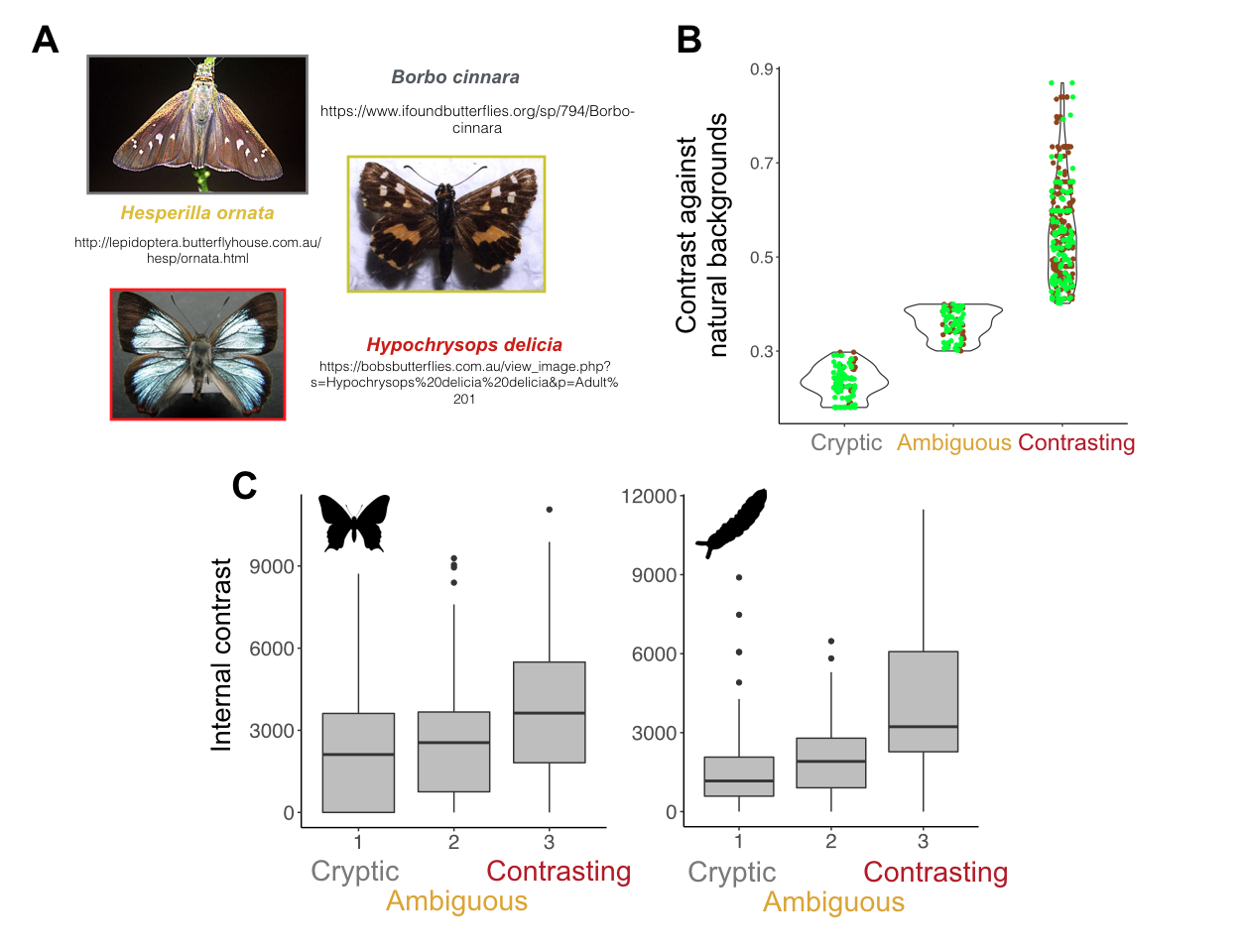


**Figure S5.** **A.** Examples of species in each anti-predator colour category, using colour code in figures B and C. **B.** Association between anti-predator colour strategy and contrast against common backgrounds (green and brown) and internal contrast. **C.** Species classified as having contrasting colourations also tend to have a higher internal contrast, a product of the combination of black with a bright colour.

**
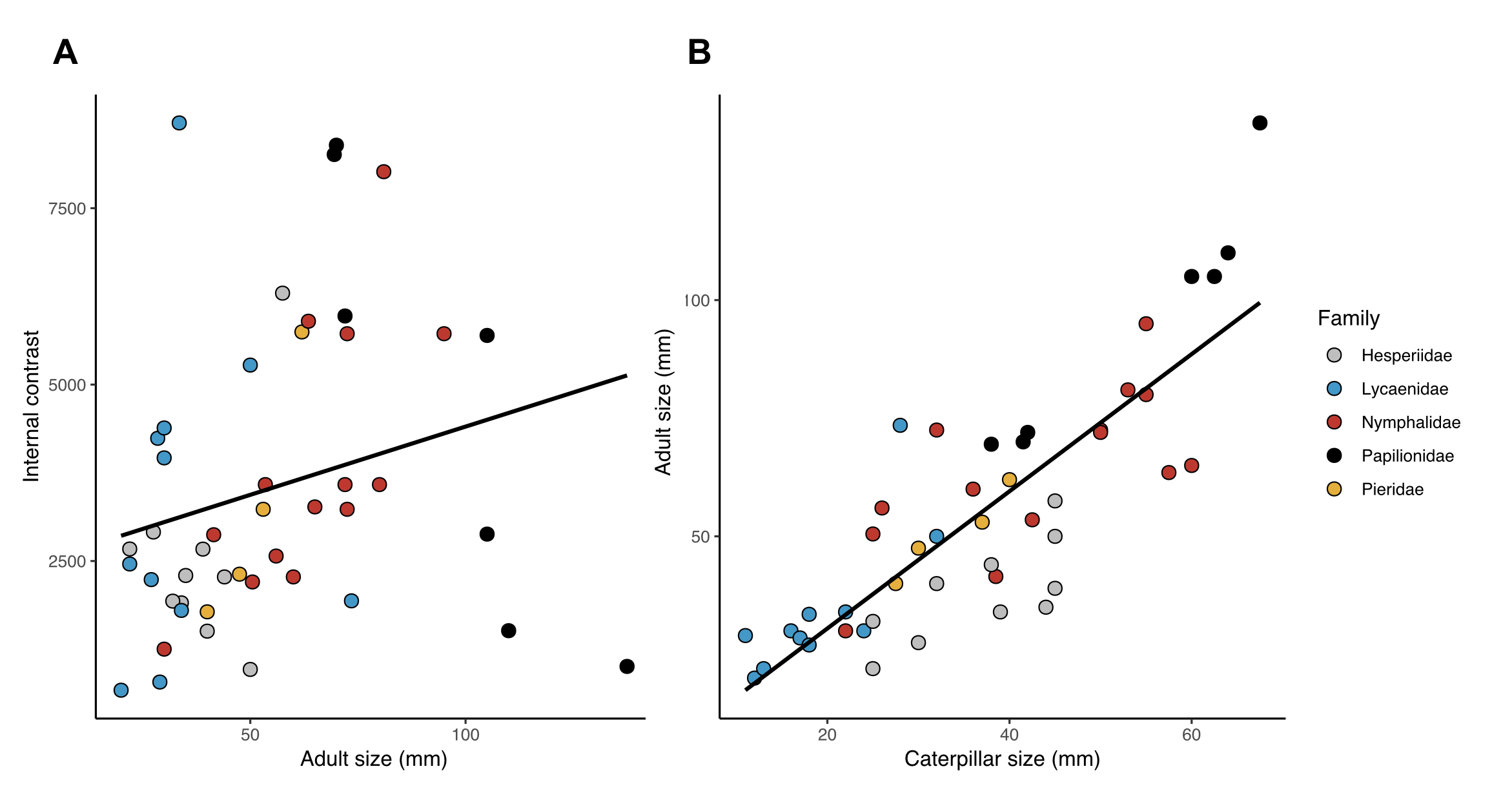
**

**Figure S6.** Association between size and internal contrast in adults (left) and caterpillar size and adult size (right).

**References**

1.

Adams, D., Collyer, M., Kaliontzopoulou, A. & Sherratt, E. (2017). Geomorph: Software for geometric morphometric analyses. R package version 3.0. 5.

2.

Drummond, A.J. & Rambaut, A. (2007). BEAST: Bayesian evolutionary analysis by sampling trees. *BMC Evol Biol*, 7, 214.

3.

Gaitonde, N., Joshi, J. & Kunte, K. (2018). Evolution of ontogenic change in color defenses of swallowtail butterflies. *Ecol Evol*, 8, 9751-9763.

4.

Heikkilä, M., Kaila, L., Mutanen, M., Peña, C. & Wahlberg, N. (2012). Cretaceous origin and repeated tertiary diversification of the redefined butterflies. *Proc. R. Soc. B*, 279, 1093-1099.

5.

Kang, C., Zahiri, R. & Sherratt, T.N. (2017). Body size affects the evolution of hidden colour signals in moths. *Proc Biol Sci*, 284.

6.

Loeffler-Henry, K., Kang, C. & Sherratt, T.N. (2019). Consistent Associations between Body Size and Hidden Contrasting Color Signals across a Range of Insect Taxa. *Am Nat*, in press.

7.

Munro, J.T., Medina, I., Walker, K., Moussalli, A., Kearney, M.R., Dyer, A.G. *et al.* (2019). Climate is a strong predictor of near-infrared reflectance but a poor predictor of colour in butterflies. *Proc R Soc Lond, Ser B: Biol Sci*, 286, 20190234.

8.

Plummer, M., Best, N., Cowles, K. & Vines, K. (2016). Package ‘coda’.

9.

Sherratt, E., Vidal-García, M., Anstis, M. & Keogh, J.S. (2017). Adult frogs and tadpoles have different macroevolutionary patterns across the Australian continent. *Nat Ecol Evo*, 1, 1385-1391.

10.

Stevens, M., PARraga, C.A., Cuthill, I.C., Partridge, J.C. & Troscianko, T.S. (2007). Using digital photography to study animal coloration. *Biol J Linn Soc*, 90, 211-237.

11.

Weller, H.I. & Westneat, M.W. (2019). Quantitative color profiling of digital images with earth mover’s distance using the R package colordistance. *PeerJ*, 7, e6398.

12.

Yu, G., Smith, D.K., Zhu, H., Guan, Y. & Lam, T.T.-Y. (2017). ggtree: an r package for visualization and annotation of phylogenetic trees with their covariates and other associated data. *Methods in Ecology and Evolution*, 8, 28-36.
